# Cultivating tobacco-free farms

**DOI:** 10.2471/BLT.22.021222

**Published:** 2022-12-01

## Abstract

A multi-agency initiative encouraging farmers to switch from tobacco cultivation to healthier and more ecologically sustainable crops is getting attention in Kenya. Gary Humphreys reports.

Kevin Genga remembers the countryside before tobacco cultivation took hold. “It was so green when I was growing up,” says the 28-year-old environmental advocate, who is based in Migori County in south-western Kenya. “Most of the county was still covered by indigenous forest.”

In fact, that ecosystem had started to change in the 1960s when British American Tobacco and other companies began pushing tobacco cultivation in the area. “They argued that tobacco growing was going to make the country richer,” Genga says.

And the argument was heard. “The perception among government officials, and farmers, was that tobacco production could generate revenue,” explains Dr Vinayak Prasad, unit head of the World Health Organization’s (WHO) Tobacco Free Initiative. “It has since become clear that this is not the case.”

Today, some 55 000 farmers grow tobacco on 13 500 hectares of land mainly in the western and south-eastern parts of the country; government data indicate that this activity contributes just 0.03% of Kenya’s GDP (gross domestic product) while tobacco farmers themselves frequently go into debt as they struggle with the razor thin margins of tobacco production.

Meanwhile, the impact on Kenya’s ecosystem has been devastating. According to Genga, in Migori County since the 1960s around 7000 hectares of forest have been cleared for tobacco planting and to fuel the fires needed for the tobacco curing process, leaving around 20 000 hectares according to Global Forest Watch, a forest monitoring app maintained by the World Resources Institute. 

Water resources have also been depleted. The precise impact of tobacco cultivation on water resources has yet to be documented and the impact of climate change in the Horn of Africa needs to be considered. For Genga, however, the anecdotal evidence is compelling. “Many of the rivers that previously fed the Migori river year-round have become seasonal,” he says, “and most of those rivers have become polluted.”

Because tobacco crops absorb more nitrogen, phosphorous and potassium than other major food and cash crops and are generally grown in monoculture without rotation, they require large amounts of chemical fertilizers and pesticides. These, along with growth regulators, end up in the ground, the aquifers and the waterways, the latter often poisoned by tobacco seedbeds which are typically located next to watercourses.

All of this has major implications for human and animal health. “Just to focus on pesticides, chronic exposure raises the risk of birth defects, benign and malignant tumours, genetic changes, blood disorders, neurological disorders, and endocrine disruption,” says Simone Saint Claire, a technical officer working on the Tobacco-Free Farms initiative at WHO in Geneva.

“We really wanted to get at supply-side issues.”

Vinayak Prasad

And, as Saint Claire is quick to point out, the crop itself is poisonous. “Exposure to wet tobacco leaves during tobacco cultivation is a particular concern,” she says. “Farmers can absorb as much nicotine as found in 50 cigarettes daily often leading to acute nicotine poisoning known as green tobacco sickness.”

The health risks are compounded in tobacco processing, notably in the curing of the leaves in huts generally located close to farmers’ homes.

Farmers like Siprone Chacha, a mother of five who used to farm tobacco in Migori County, and recalls the practice of tobacco curing with a shudder. “We had to cure the leaves non-stop for three days,” she says. “That meant tending the fires and adding firewood and checking the temperature and the leaves’ condition throughout the night and all the time breathing in the smoke.” Once the leaves had been cured, they were stored in the house, exposing Chacha and her family to tobacco dust.

Because they push out more nutritious crops, tobacco also impacts nutritional security, while the labour-intensive nature of tobacco cultivation and processing impacts education. A study carried out in the Nyanza Province of Kenya found that tobacco growing required an average of 220 days of labour, far higher than for other crops such as soya bean which requires just over 50. Because of this, farmers tend to enlist the support of family members, including children who miss school as a result.

To tackle this constellation of problems, WHO joined forces with the World Food Programme (WFP), the Food and Agriculture Organization and the Kenyan government to launch the Tobacco-Free Farms project in March 2022. Nine months later the project is bearing fruit.

For WHO, the project represents a significant new departure in its tobacco control work. “As an organization we have tended to focus on demand reduction,” says Prasad. “In this case we really wanted to get at supply-side issues while also mitigating the harmful impacts of tobacco cultivation on human and animal health and the environment.”

According to Prasad, Kenya was considered a suitable entry point because of strong government support for the project. Kenya was one of the first countries to ratify the legally binding WHO Framework Convention on Tobacco Control in 2004 and has been proactive in implementing and championing strong and effective tobacco control measures.

Core to the Tobacco-Free Farms project is the presentation of a viable alternative to tobacco farming. “It’s not enough to explain to farmers the ways in which tobacco farming is bad for them – they already know that. It has to work from a business point of view,” says Calvince Onyango, the WHO Project Officer on the alternative livelihoods to tobacco growing project in Migori County.

Siprone Chacha is a case in point. Like many other farmers, she was tempted into tobacco cultivation by the prospect of increasing her income. However, she found that by the time she had cut down trees, cleared land, planted the crop, paid for the pesticides and the fertilizer, and then carefully nurtured the crop to maturity it was hard to make a profit.

On top of everything else, the buyer with whom she had a contract did not accept all of her leaves. “If the leaf was not perfect, he would not take it which meant that I had to dump the unsold crop on my land which was poisoned by the nicotine,” she recalls.

It was for these and the other compelling health and ecosystem reasons that Chacha was ready to consider alternative crops.

But it was still not an easy sell for Onyango and his colleagues. “The truth is the land is impoverished by tobacco cultivation and it can take many years to get it back to the state of health required to grow other crops,” he says.

“I had to dump the unsold crop on my land.”

Siprone Chacha

A big question for Chacha was how to get through that transition period. Key to meeting that challenge was planting the right crop. Chacha decided on soya bean, but most Migori County farmers have planted high-iron beans, which are easy to grow, feed the land and the farmers’ families and only take 60 days to come to maturity.

The collaborating United Nations (UN) agencies and the Kenyan government have provided seeds and fertilizers to farmers to grow Nyota beans but, as Prasad is at pains to point out, the Tobacco-Free Farms initiative relies heavily on galvanizing producers and developing markets rather than creating passive recipients of aid.

According to Prasad, key to achieving that aim is the Farm to Market Alliance initiative being implemented by the Cereal Growers Association on behalf of the WFP. The initiative supports farmers through a network of Farmer Service Centres that aim to enhance farmers’ productivity, increase market linkages and encourage private sector investment.

“They work alongside the WFP, but basically act more like a private sector player, bringing together farmers and buyers and encouraging the development of very consolidated, very empowered farmers’ groups,” Prasad explains.

The WFP has also supported farmers in transition from tobacco to other crops by providing a ready market for their harvest as a backstop through a regional procurement scheme.

Notwithstanding these different initiatives, some farmers will still need financial help. According to Prasad discussions are ongoing with the UN Capital Development Fund, one of the few UN agencies that can give money to the private sector. “We are hoping to get them to engage with microfinance institutions to ensure effective distribution of funding,” he says.

Thus far Prasad is excited by the results. “We expected a couple of hundred farmers starting with us on this journey, but farmers are seeing the project as a real opportunity to get out of tobacco and over a thousand have already joined.”

Prasad is also pleased to see health benefits starting to feed through. “We are seeing anecdotal evidence of farmers’ health improving as well as increased school attendance from children previously working on the farms,” he says.

According to Prasad there are plans to pass on the experience and knowledge gained from the Tobacco-Free Farms project to Kenyan tobacco farmers outside Migori County, but he is already looking at neighbouring Zambia to replicate the model. “We want to apply the lessons learned in Kenya in another country,” he says. “That’s the plan.”

**Figure:**
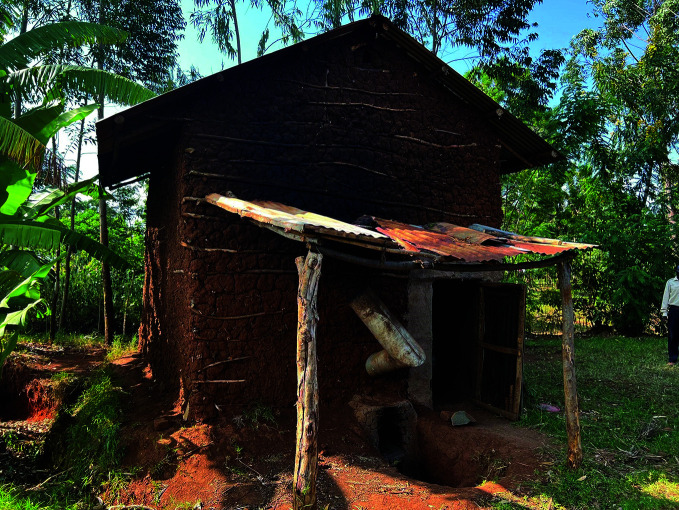
A tobacco curing hut in Migori County, Kenya.

WHO/Ash Appleton

**Figure:**
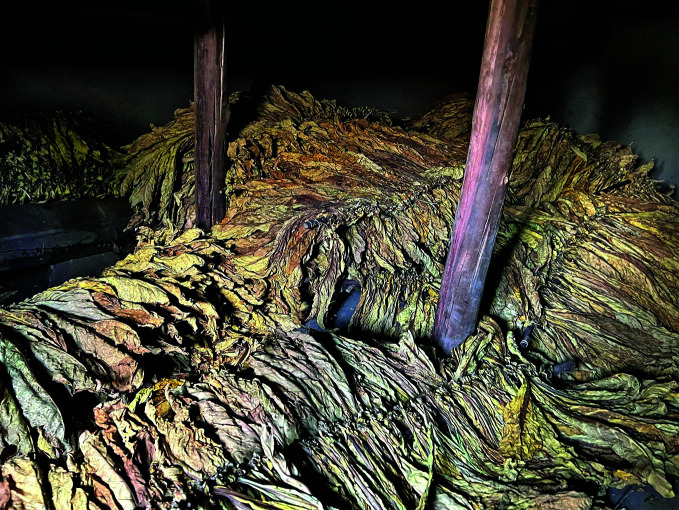
Tobacco leaves drying in Migori County, Kenya.

WHO/Ash Appleton

